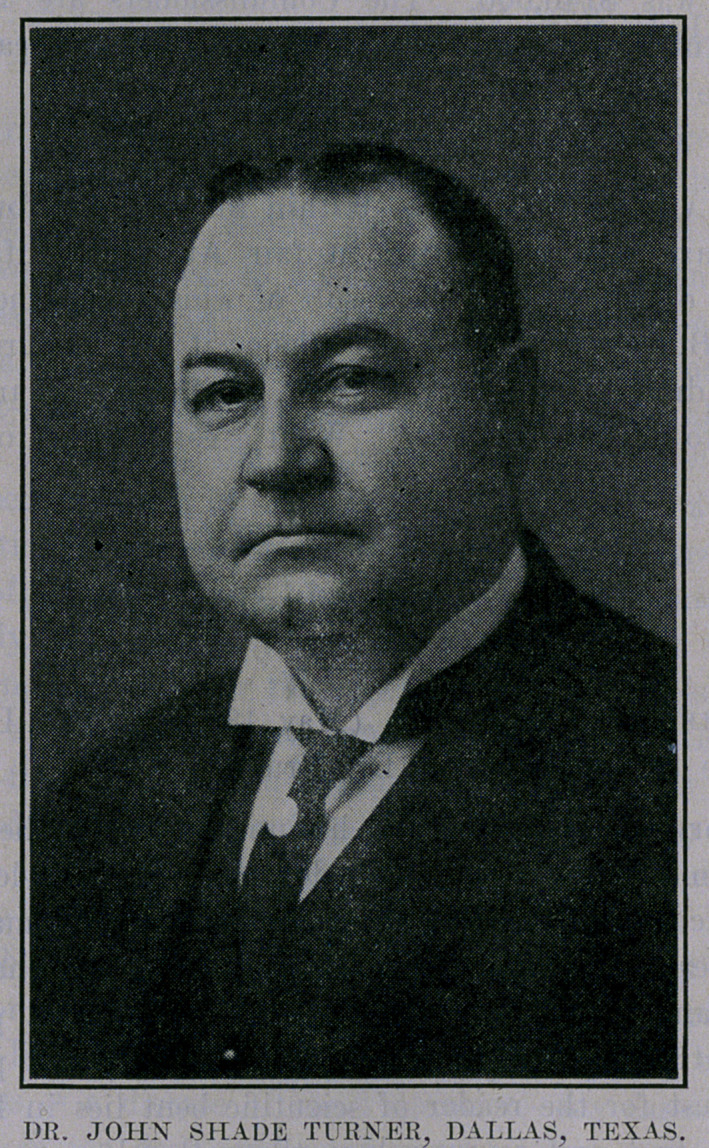# News and Miscellany

**Published:** 1912-06

**Authors:** 


### News and Miscellany.

THE SCIENTIFIC AMERICAN (May 18th) says of Dr. Daniel's book, "The Strange Case of Dr. Bruno":

"The story of Dr. Bruno and his uncanny researches is far removed from the present-day orthodox novel with its love and the petty obstacles thereto that, swept away, leave the man and the woman in each other's arms at the end of the last chapter. The real and fancied relationships which make the plot possible become so intricate as to be almost bewildering. But perhaps the main interest for the reader of scientific bent lies in Dr. Bruno's attempt to find and to reproduce the molecular construction of that secretion which the mud-wasp injects into her victims, causing an almost complete suspension of vital functions without actually destroying life. Not a few of humanity's greatest physiological, social and moral problems are brought into the limelight, and examined in a virile manner from unusual points of view. The tale is at the same time instructive and absorbing, and its most extreme theorems are advanced with so matter-of-fact an attention to detail that their plausibility seems beyond question.

THE SAGE OF BUFFALO.—The JOURNAL regrets to learn that Dr. Sam R. Burroughs, ex-President of the State Medical Association, *et cetera,* is in rather bad health, and has been compelled to give up practice altogether—after forty-ve years of strenuous activity. He was not able to attend the Waco meeting. Dr. Burroughs is one of the great men of the Texas profession, and is more beloved and esteemed perhaps than any member now living.

---

DR. JOHN SHADE TURNER, DALLAS, TEXAS.

President-elect Texas State Medical Association.

Born in Sumpter county, Georgia, February 16, 1866. His parents, Green B. and Mattie (Scott) Turner, removed from the old home to Johnson county, Texas, where he was reared. Educated in the public schools and in Professor Long's Academy, Cleburne, Texas. Graduated from the Louisville Medical College, Louisville, Kentucky, with the class of 1889, after having practiced for a time upon a State certificate.

Married to Miss Mattie R. Hightower, March 12, 1885,—to this union three daughters and two sons were born, the sons dying in infancy.

After graduation Dr. Turner located at Stephenville, Texas, where he practiced for a few years; later going to Granbury, from which place in January, 1897, he was appointed Assistant Superintendent to the Southwestern Insane Asylum, San Antonio, Texas, serving in that capacity for three years and a half. In June, 1900, he was appointed to the Superintendency of the North Texas Hospital for the Insane, Terrell, Texas, the second largest institution of its kind in the South. After serving as Superintendent of the last named Hospital for six and a half years, Dr. Turner voluntarily retired from the State's service,—the same position being again tendered to him,—thus completing a continuous public service of ten years.

In February, 1907, Dr. Turner removed to Fort Worth and opened the Arlington Heights Sanitarium, which he conducted for two years, disposing of the institution and locating in Dallas, his present home, in order to devote his entire time to consultation work along the line of mental and nervous diseases, and to become the Medical Director of the Southland Life Insurance Company.

Dr. Turner has served in various capacities in furthering the principles of organized medicine in the State. He began as Secretary of the Hood County Medical Society. Later was President of the Kaufman County Medical Society; member of the House of Delegates from Kaufman county; President of the Tri-State Medical Association (now the Southwestern Medical Association); member of the Board of Counsellors in the American Medico-Psychological Association; member of the Legislative and of Care and Treatment of the Insane Committees, at different times, from the Texas State Medical Association, and Delegate to the A. M. A.

Dr. Turner is at this time President of the Texas Medical Directors' Association; President of the Dallas City Medical and Surgical Society; President of the Board of Censors of the Dallas County Medical Society; member of the Board of Health of the city of Dallas; member of the Board of Counselors of the Bureau of Scientific Temperance Investigation and of Scientific Instruction in Schools and Colleges, for the World's and National Organizations of the Woman's Christian Temperance Union; and Professor of Mental and Nervous Diseases, Baylor Medical College.

---

When there is disagreement between the pulse and temperature, the pulse must be regarded as of the greater importance.—*American Journal of Surgery.*

## Figures and Tables

**Figure f1:**